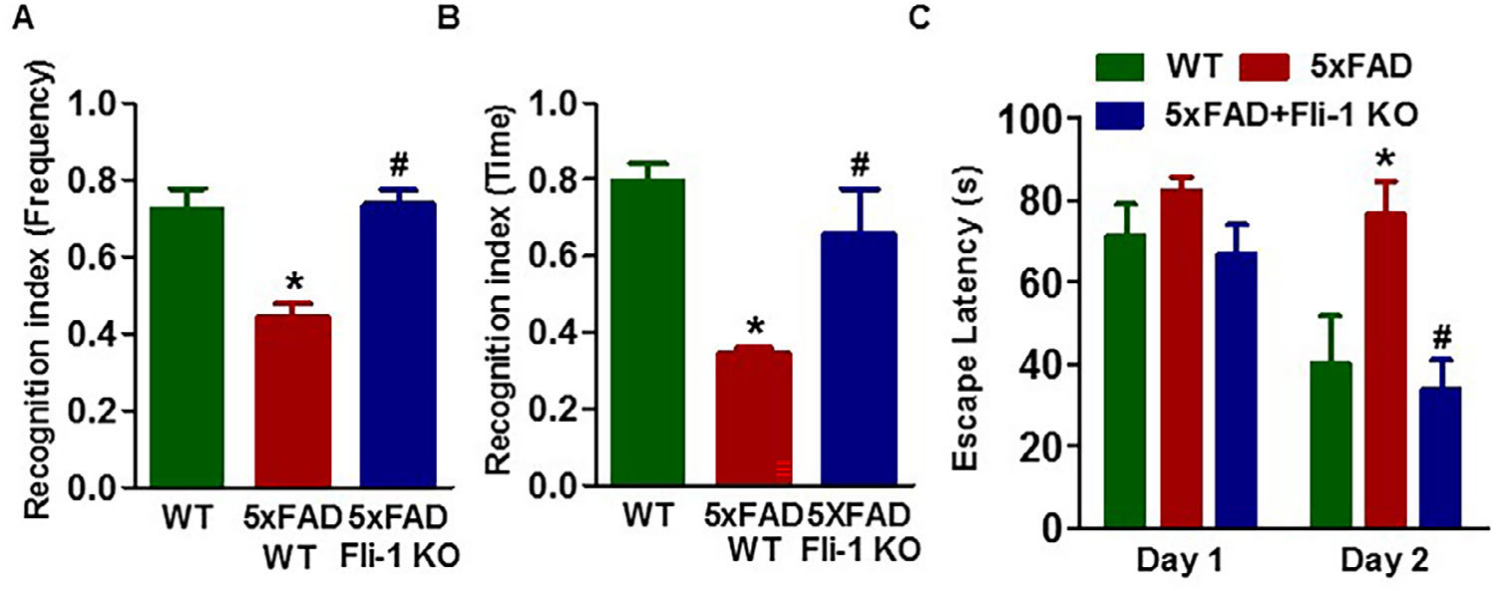# Unveiling the role of Fli‐1 in controlling astrocyte dysfunction and transformation in Alzheimer’s disease

**DOI:** 10.1002/alz.086131

**Published:** 2025-01-03

**Authors:** Pengfei Li, Andrew Goodwin, Perry Halushka, Hongkuan Fan

**Affiliations:** ^1^ Medical University of South Carolina, Charleston, SC USA

## Abstract

**Background:**

Alzheimer’s disease (AD) is associated with cognitive impairment and neuro‐inflammation. Dysregulated activation of microglia and astrocytes induces neuro‐inflammation, and reactive astrocytes have been classified into A1 neurotoxic and A2 neuroprotective phenotypes. A1 astrocytes are induced by activated neuro‐inflammatory microglia via secreting IL‐1α, TNFα and C1q, and contributing to inflammation and neuronal cell death. However, the processes that govern astrocyte transformation and their role in AD development remains unclear. Increased friend leukemia virus integration 1 **(**Fli‐1) levels were observed in the hippocampus of brain tissue from AD patients and 5xFAD mice, a murine model of AD. While inhibition of Fli‐1 reduced pericyte loss, Aβ deposition and BBB dysfunction in the 5xFAD mice. However, Fli‐1’s specific role in astrocyte activation in AD remains unexplored.

**Method:**

Fli‐1 levels and A1 phenotype astrocyte number were detected in brain tissues from WT mice, 5xFAD mice and inducible Fli‐1 KO 5xFAD mice. Control or Fli‐1 antisense oligonucleotide Gapmers were injected into the hippocampus of 5xFAD mice at 3 and 4.5 months of age. A1 phenotype astrocyte number, inflammatory mediators and cognitive deficits were evaluated at 6 months of age. Cultured mouse astrocytes were transfected with control or Fli‐1 Gapmers for 24 h and followed stimulation with IL‐1α, TNFα and C1q for another 24 h. The expression levels of Fli‐1 and inflammatory mediators, and A1 phenotype astrocyte number were determined.

**Result:**

Fli‐1 levels are higher in the astrocytes in hippocampus of 5xFAD mice, and corresponded with increased astrocyte activation, A1 astrocyte number, inflammation and cognitive impairment. While 5xFAD mice with inducible Fli‐1 KO exhibited decreased astrocyte activation, attenuated inflammation, and mitigated cognitive impairment. Knockdown of Fli‐1 in cultured mouse astrocytes with Fli‐1 Gapmers suppressed activated microglia‐induced astrocyte activation, and inhibited IL‐1α, TNFα and C1q‐indcued A1 astrocyte transformation *in vitro*. Moreover, injection of Fli‐1 Gapmers into the hippocampus of 5xFAD mice significantly decreased Fli‐1 levels and reduced A1 astrocyte number.

**Conclusion:**

The elevated Fli‐1 levels contribute to astrocyte dysfunction and A1 astrocyte transformation in AD. Targeting Fli‐1 could be a therapeutic approach to prevent or mitigate AD‐induced astrocyte dysfunction and cognitive impairment, offering potential clinical applications.